# Visits to Private Asylums

**Published:** 1903-11-14

**Authors:** 


### VISITS TO PRIVATE ASYLUMS.

By Our Rambling Reporter.

### THE GROVE, OLD CATTON, NORWICH.

From the Thorpe Station, Norwich, this asylum is distant about two miles. The electric trams run to about half of this distance, and from the tram terminus the walk is along a quiet country road. The house is very prettily situated among trees, and is secluded rather than shut in. About ten acres of land surround it, and some of this land is laid out in lawns, walks, and flower gardens which are always accessible to and used by the patients. The whole place is distinctly of the private mansion type, indeed we understand it was at one time occupied by a Norfolk County family; and although it has been added to as occasion required the alterations have been carried out in such a manner that its original character has been preserved. The asylum is managed and conducted just like a country house, and among other things we noted is absence of locked doors, an example we should like to see followed in all the smaller private asylums in England. The house is licensed for 21 patients, and only ladies are received. The license is about 50 years old, and has been held about eight years by the present proprietors, who are Dr. and Mrs. Osborne and Miss McLintock. Many of the rooms are large and are very nicely fitted up and furnished. It is the admirable practice of the licensees to take their meals with the patients, and this has always a strong tendency to preserve the home-like life of such an establishment. In the ordinary acceptation of the term there are no dormitories, but there are two or three of the rooms which have three beds each, and this is a desirable arrangement to the extent it is carried out at The Grove, because there are always some of the more nervous patients who like the cheerfulness of sharing a room with others. Private sitting-rooms are provided for those who may want privacy. It would seem that the recoveries constitute a high percentage of the admissions, and it may be added that deaths among the patients are very rare. The fees range from two and a half guineas a week upwards, according to the accommodation required.

